# MFG-E8 has guiding significance for the prognosis and treatment of sepsis

**DOI:** 10.1038/s41598-022-25601-8

**Published:** 2022-12-03

**Authors:** Wei Wu, Jing Wang, Jingdi Chen, Jiaming Lu, Yaojia Lao, Kai Huang, Jun Lin

**Affiliations:** 1 Department of Critical Care Medicine, Renmin Hospital of Wuhan University, Wuhan, Hubei People’s Republic of China; 2 Department of Gastroenterology, Renmin Hospital of Wuhan University, Wuhan, Hubei People’s Republic of China; 3 Department of Orthopedics, The Airborne Military Hospital, Wuhan, Hubei People’s Republic of China; 4 Department of Gastroenterology, Zhongnan Hospital of Wuhan University, Wuhan, Hubei People’s Republic of China

**Keywords:** Cell death, Infectious diseases, Biomarkers

## Abstract

Sepsis remains a significant clinical challenge. Ferroptosis is involved in the pathogenesis of sepsis. Ferroptosis is associated with oxidative stress, and excessive oxidative stress is suppressed by milk fat globule epidermal growth factor 8 (MFG-E8) under various conditions. However, the role of MFG-E8 in sepsis-induced ferroptosis and oxidative stress is still unclear. First, we collected blood samples from patients with sepsis and detected the expression of serum MFG-E8. Then, the relationship between serum concentrations of MFG-E8 and disease severity was detected. Finally, the effects of MFG-E8 treatment on ferroptosis and oxidative stress in the livers of septic mice were determined. The expression of serum MFG-E8 in healthy subjects was notably higher than that in septic patients. In addition, when nonsurvivors and survivors of sepsis were compared, MFG-E8 levels were considerably lower in the former. The ROC curve for MFG-E8 was also generated. The area under the curve for MFG-E8 was 0.768 (95% confidence interval [CI] 0.627–0.909, p = 0.003). The patients were separated into two groups based on the MFG-E8 cut-off value of 3.86 ng/mL. According to the Kaplan‒Meier survival analysis, patients with low MFG-E8 levels had a significantly decreased 28-day survival rate compared with patients with high MFG-E8 levels. High MFG-E8 levels were substantially related to a decreased risk of death, as demonstrated by the Cox proportional hazard model that we utilized. In addition, compared with sham mice, septic mice exhibited liver and kidney damage, and MFG-E8 may have protective effects. The survival study indicated that MFG-E8 could effectively improve the survival rate of septic mice. Treatment with MFG-E8 suppresses oxidative stress and ferroptosis in the livers of septic mice. Serum MFG-E8 levels are lower in septic patients and are negatively related to disease severity. Treatment with MFG-E8 suppresses oxidative stress and ferroptosis in the livers of septic mice, contributing to significantly improved survival in septic mice. These findings showed that MFG-E8 could be a new sepsis predictive biomarker. MFG-E8 may have therapeutic potential in the treatment of sepsis.

## Introduction

Sepsis is a life-threatening disease caused by an imbalanced inflammatory response. Although survival from sepsis has improved, sepsis still results in a 27% mortality rate and long-term physical and cognitive disabilities^1^. Considering the complex and broadly activated inflammatory state, researchers have sought drugs that can thoroughly regulate the immune response and attenuate organ dysfunction. However, thus far, their results have only yielded limited benefits^2^. Additional studies are required to explore more effective treatments.

Milk fat globule epidermal growth factor 8 (MFG‐E8) is a glycoprotein secreted by activated macrophages and immature dendritic cells and is widely expressed in various organs^3–6^. However, it is uncertain whether MFG-E8 is expressed in the serum of septic patients. MFG-E8 is highly appreciated in inflammatory diseases, including sepsis, colitis, ischaemia and reperfusion-induced intestinal injuries, for its scavenging role and anti-inflammatory properties^7–9^. MFG-E8 performs an anti-inflammatory function mainly by enhancing the clearance of apoptotic cells, but the other mechanisms associated with its role have not been clearly revealed^10,11^. Neuroinflammation research demonstrates the involvement of MFG-E8 in antioxidant effects^12^. The role of MFG-E8 in septic oxidant responses is still unclear.

Recently, ferroptosis has been verified to take part in the pathological processes of various diseases^13–15^, including organ injury and oxidative stress in sepsis. Some studies have indicated that inhibiting ferroptosis can protect against organ injury caused by sepsis^16,17^. However, the association between MFG-E8 and ferroptosis in sepsis has not yet been confirmed. According to earlier research, recombinant mouse MFG-E8 reduces organ injury^18,19^. Thus, we inferred that MFG-E8 treatment may regulate ferroptosis and protect organ function in sepsis. Hence, the effects of MFG-E8 administration on ferroptosis and oxidative stress in the livers of septic mice were determined.

To explore MFG‐E8’s role in sepsis and investigate the relationship between MFG-E8 and oxidative stress and ferroptosis, we first collected blood samples from patients with sepsis and assessed the relationship between serum MFG‐E8 concentrations and the severity of the disease, as well as the effects and putative mechanisms of MFG-E8 therapy in experimental sepsis models. The results demonstrate the important role of MFG‐E8 in sepsis.

## Methods

### Patients

One hundred septic patients (age > 18 years) in Renmin Hospital of Wuhan University between August 2020 and December 2020 were involved in this observational study. According to the Sepsis-3 criteria, blood culture results and organ dysfunction are considered to be diagnostic criteria for sepsis [(1) suspected infection and (2) Sequential Organ Failure Assessment (SOFA) score ≥ 2], while patients with malignant tumours were excluded. Blood samples of septic patients were collected within 24 h of hospital admission. The study was authorized by the Ethics Committee of Renmin Hospital of Wuhan University (approval no. WDRY2020-K221), and all informed consent forms were signed by the participants. The Declaration of Helsinki and the principles for Good Clinical Practice were followed during the study’s execution.

### Experimental models

The Experimental Animal Center of Renmin Hospital of Wuhan University provided male adult C57BL/6J mice (20–26 g). The caecal ligation puncture (CLP) model was used to induce polymicrobial sepsis as previously discussed^20^.

A mixture of ketamine (100 mg/kg) and xylazine (10 mg/kg) was used to anaesthetize mice intraperitoneally, the abdomen was disinfected, and an incision was made to expose the caecum. The caecum was exteriorized, and then silk thread was used to ligate under the ileocaecal valve. Then, a 23-gauge needle was used to puncture the distal end of the ligation twice, and the tissue was gently squeezed to extrude a small amount of stool. The abdomen was then closed. In the sham operation group, only laparotomy was performed, and the caecums were neither punctured nor ligated. The mice were then resuscitated with prewarmed normal saline (0.05 mL/g body weight) injected subcutaneously. The mice were eventually returned to their cages and given adequate food and water.

At 5 h after CLP, recombinant mouse MFG-E8 (rmMFG-E8 (Cat. No.: 2805-MF-050; R&D Systems, Minneapolis, MN) 0.1 mL, 20 μg/kg) was administered intravenously. The control group was injected with an equal volume of normal saline. The animals were given unlimited access to food and water up to the scheduled intervention time. Some mice were used for Kaplan–Meier survival analysis, and others were euthanized at 12 h or 24 h after CLP according to the purpose of the experiment. Blood and liver tissue samples were collected for various ex vivo analyses. The study was conducted according to the ARRIVE guidelines and was approved by the Institutional Animal Care and Use Committee of the Ethics Committee of Renmin Hospital of Wuhan University (approval no. WDRY2020-K221).

### Enzyme-linked immunosorbent assays

MFG-E8 commercial ELISA kits (E-EL-H2063c, Elabscience Biotechnology Co., Ltd., Wuhan, China), TNF-α ELISA kits (CSB-E04741m, Cusabio Biotech Co., Ltd., Wuhan, China) and Interleukin (IL)-6 ELISA kits (CSB-E04639m, Cusabio Biotech Co., Ltd., Wuhan, China) were used to measure serum MFG-E8, TNF-α, and IL-6 levels following the manufacturers’ instructions.

### Malondialdehyde, glutathione, and iron assays

A lipid peroxidation (malondialdehyde (MDA)) assay kit (S0131S, Beyotime, China), a total glutathione assay kit (S0052, Beyotime, China), and an iron assay kit (BC1735, Solarbio Science & Technology Co., Ltd. Beijing, China) were used to evaluate the lipid peroxidation product MDA and reduced glutathione (GSH) levels and ferrous iron (Fe2+) concentrations following the manufacturers’ instructions.

### RNA extraction and quantitative real-time RT-PCR

In this study, liver tissue samples from septic mice were obtained, TRIzol reagent was used to extract total RNA, and qPCR was conducted as described previously^21^. The specific primers for mouse IL-6, TNF-α, IL-1β, CXCL-1 and GAPDH are shown in Supplementary Table 1.

### Analysis of organ injury markers

Blood samples were centrifuged at 3000×*g* for 10 min to collect serum and were then immediately analysed for organ injury parameters. Aspartate aminotransferase (AST, SEB214Mu, Cloud-Clone Corp., formerly USCN Life Science Inc., Wuhan, China), alanine aminotransferase (ALT, SEA207Mu, Cloud-Clone Corp.), lactate dehydrogenase (LDH, SEB864Mu, Cloud-Clone Corp.), and creatinine (Cr, CEV806Ge, Cloud-Clone Corp.) levels were assessed using commercial assay kits.

### Western blot analysis

Liver tissue samples from septic mice were obtained and ground, and protein extraction and western blot analysis were performed as described previously^21^. The primary antibodies included anti-GPX4 antibody (ab125066, 1:3000) from Abcam (Cambridge, UK) and anti-GAPDH (AF1186, 1:3000) from Beyotime Biotechnology (Shanghai, China). ImageJ software was applied to quantify the band intensity, and the ratio of the target protein to GAPDH was used to display the results.

### Statistical analysis

One-way ANOVA or T test was used to compare the normally distributed data, which are presented as the mean ± standard deviation. Nonnormally distributed data are expressed as medians and interquartile ranges (IQRs) and were compared by the Kruskal‒Wallis/Mann‒Whitney U test. A test for the homogeneity of variance was conducted, and nonparametric tests were utilized when the variances were uneven. The counting data are expressed as rates (%), and the comparison between groups was tested by χ^2^. The Spearman correlation coefficient was used to analyse correlations. ROC curves were used to analyse the diagnostic efficacy of MFG-E8. To assess the relationships between each variable and mortality, we employed Cox’s proportional hazard model to compute the hazard ratio (HR). Survival analyses were statistically analysed by the log‐rank test. IBM SPSS 26.0 software was used to analyse relevant data, apart from the Harrell C-index, which was performed using STATA 15 software. *p* values < 0.05 were considered statistically significant.

### Ethics approval and consent to participate

Ethical committee approval was required for this study.

## Results

### Serum MFG-E8 levels are decreased in septic patients and are negatively correlated with disease severity

**Figure 1:**
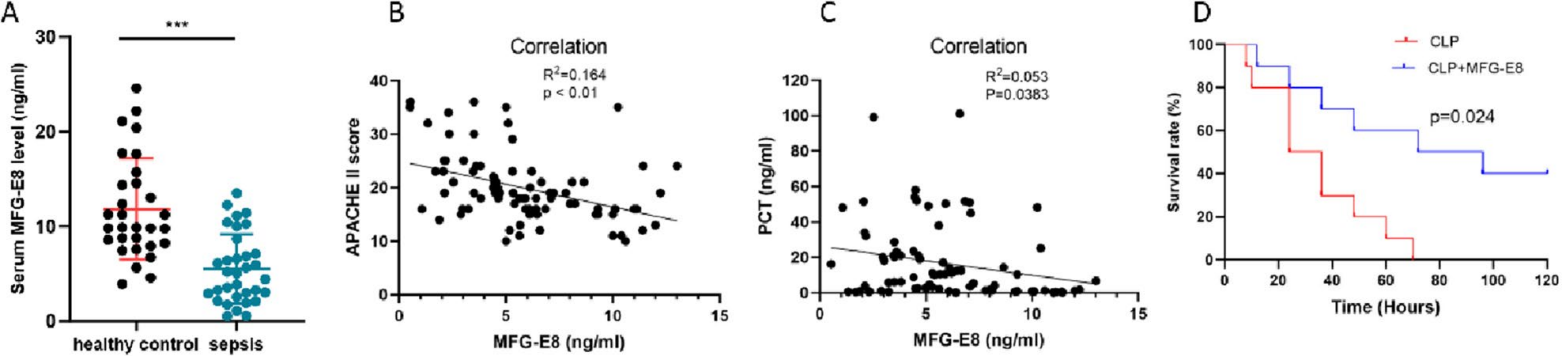
Serum MFG-E8 levels are decreased in patients with sepsis compared with healthy controls and are negatively correlated with disease severity. (**A**) Serum MFG-E8 levels in 100 septic patients and 30 normal individuals. Mean ± SEM, ***p < 0.001. (**B**) Correlation analysis of the Acute Physiology and Chronic Health Evaluation (APACHE) II score and serum MFG-E8 levels of septic patients. p = 0.0001. (**C**) Correlation analysis of procalcitonin (PCT) and serum MFG-E8 levels of septic patients. p = 0.0383. (**D**) MFG-E8 improves the survival in cecal ligation and puncture (CLP)-induced sepsis.

From August 2020 to December 2021, 100 septic patients and 30 healthy subjects were included in the study. The baseline characteristics of the patients and controls are shown in Supplementary Table 2. The differences in age, sex and biochemical results were not statistically significant, but the serum MFG-E8 levels in healthy subjects (11.74 ± 5.44 ng/mL) were apparently higher than those in septic patients (5.60 ± 3.80 ng/mL) (Supplementary Table 2, Fig. 1A). In addition, the levels of MFG-E8 were negatively correlated with the Acute Physiology and Chronic Health Evaluation II (APACHE II) score (Fig. 1B) and procalcitonin (PCT) levels (Fig. 1C) in septic patients. The APACHE II score predicts the hospital mortality of patients, and a higher score indicates a worse outcome of critical patients. PCT is a diagnostic marker for severe sepsis^22^. MFG-E8 may be a supporting factor for assessing the severity and poor prognosis of sepsis.

To explore the effect of MFG-E8 on the prognosis of septic patients, the patients were divided into survivor and nonsurvivor groups. Table 1 shows the study cohort’s baseline characteristics. The most common infectious sources were the lung, intraabdominal cavity and urinary tract. Hypertension, diabetes mellitus and chronic kidney disease were common comorbidities in the two groups. Compared with the survivor group, the nonsurvivor group had lower levels of serum MFG-E8 (6.2 ± 3.6 µg/mL vs. 3.2 ± 1.9 µg/mL, p < 0.001). PCT levels were 22.3 ± 7.1 ng/mL in nonsurvivors and 6.8 ± 4.73 ng/mL in survivors (p = 0.017). Compared with survivors, nonsurvivors had higher APACHE II scores and SOFA scores (p < 0.001). There was no statistically significant association between MFG-E8 levels and other factors, such as age (p = 0.65), sex (p = 0.72), white blood cells (WBCs) (p = 0.43), ALT (p = 0.067), platelet number (p = 0.064), Cr (p = 0.64), albumin (p = 0.76) and natriuretic peptide (p = 0.073).

**Table 1:** Patient characteristics of survivors and non-survivors.

Variables	Survivors (N = 89)	Non-survivors (N = 11)	*p* value
**Demographics**			
Age (years, Mean ± SD)	62.6 ± 15.3	65.7 ± 17.0	0.65
Gender(male/female)	43/46	4/7	0.72
**Comorbidities (%)**			
Hypertension	55 (61.8)	7 (63.6)	0.63
Diabetes mellitus	41 (46.1)	5 (45.5)	
Chronic kidney disease	25 (28.1)	3 (27.3)	
Cirrhosis	6 (6.7)	1 (9.1)	
**Infectious source (n, %)**			
Lung	48 (53.9)	6 (54.5)	0.73
Intraabdominal	31 (34.8)	4 (36.4)	
Urinary	7 (7.9)	1 (9.1)	
Skin	2 (2.3)	0	
Other	1 (1.1)	0	
**Laboratory results (mean ± SD or median, interquartile range)**			
WBC (× 10^9^/L)	11.5 ± 4.1	13.5 ± 2.8	0.43
Neutrophils (%)	84 ± 5.7	86.2 ± 6.5	0.35
Platelet count (× 10^9^/L)	157.4 ± 56	189.4 ± 62	0.075
ALT (U/L)	67 (7–5380)	95 (2–6001)	0.067
TBIL (μmol/L)	24.1 (3.99–290)	35.2 (3.99–298)	0.48
Cr (μmol/L)	80.5 (17–690)	108 (28–827)	0.64
ALB (g/L)	32.5 ± 3.9	30.7 ± 6.4	0.76
NT-proBNP (pg/mL)	8073 (7–52,663)	5746 (28–49,086)	0.073
cTnT (pg/mL)	478 (7–5,139,000)	260 (7–7,240,000)	0.47
PCT (ng/mL)	6.8 ± 4.73	22.3 ± 7.1	**0.017**
MFG-E8 (ng/mL)	6.2 ± 3.6	3.2 ± 1.9	** < 0.001**
**Clinical scoring, points, median (IQR)**			
APACHE II score	16.5 ± 4.9	24.3 ± 7.1	** < 0.001**
SOFA score	6 (8)	11 (9)	** < 0.001**

*WBC* white blood cells, *cTnT* cardiac troponin T, *NT-proBNP*, NT-proB-type Natriuretic Peptide, *ALT* alanine transaminase, *TBIL* total bilirubin, *Cr* creatinine, *ALB* albumin, *PCT* procalcitonin, *MFG-E8* milk fat globule epidermal growth factor 8, *APACHE II score* Acute Physiology and Chronic Health Evaluation II score, *SOFA score* Sequential Organ Failure Assessment Score.

Significant values are in bold.

In conclusion, we found that serum MFG-E8 levels are decreased in septic patients and negatively correlated with disease severity.

### Predictive value of MFG-E8 for 28-day survival

**Figure 2:**
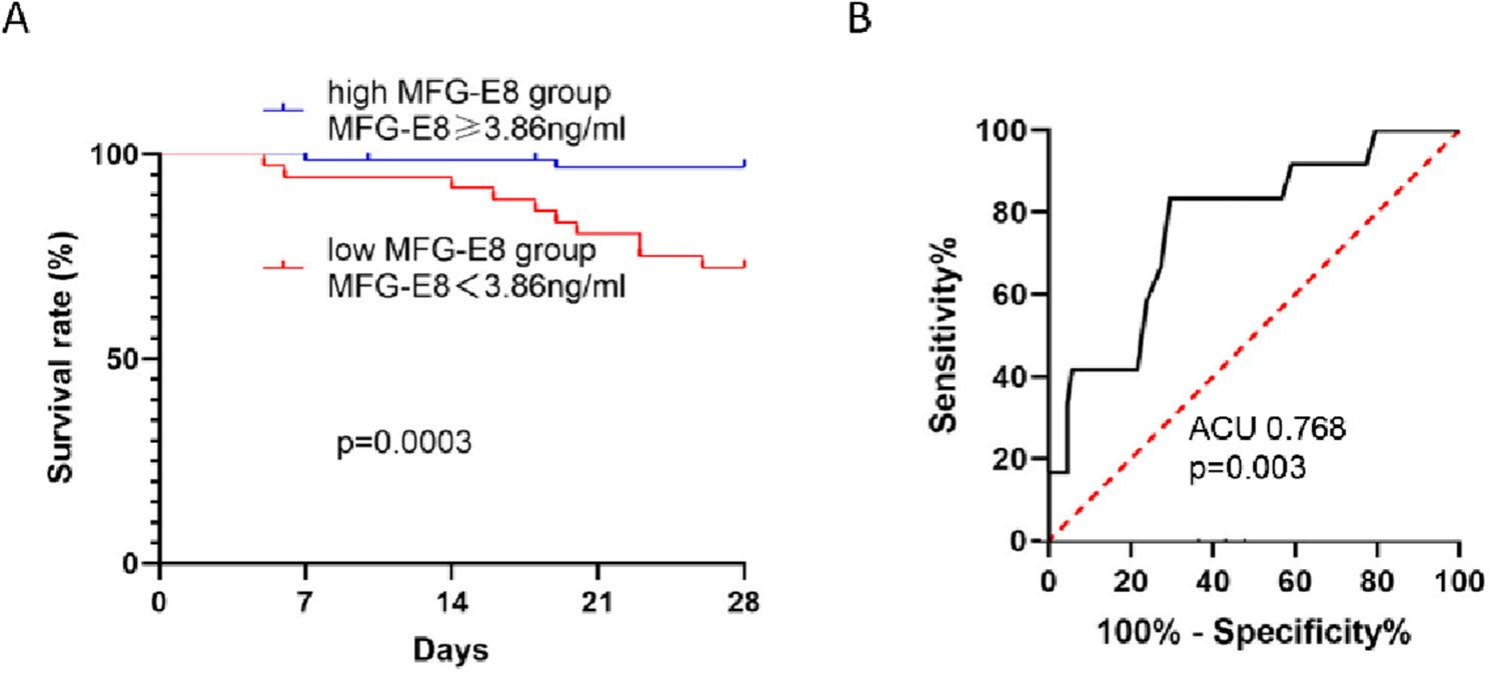
The ROC curves of predicting for 28-day survival and Kaplan–Meier estimator analysis of MFG-E8 for 28 day survival. (**A**) Kaplan–Meier estimator analysis of MFG-E8 for 28-day survival. The cut off value of MFG-E8 for the estimated 28-day mortality of patients with sepsis was 3.86 ng/mL. (**B**) The ROC curves of MFG-E8 for the predicting 28-day mortality in patients with sepsis.

We calculated the ROC curve for MFG-E8-predicted mortality during the 28-day follow-up period. The area under the curve was 0.768 (95% confidence interval [CI] 0.627–0.909, p = 0.003, Fig. 2A). According to the cut-off value of 3.86 ng/mL of MFG-E8, with a sensitivity and specificity of 83% and 71%, respectively (Supplementary Table 3), all patients were separated into two groups. According to Kaplan‒Meier survival analysis, patients with low MFG-E8 levels had a significantly lower 28-day survival rate than patients with high MFG-E8 levels (p = 0.0003, Fig. 2B).

To assess the relationships between each variable and mortality, we utilized Cox’s proportional hazard model (Table 2). Significantly decreased mortality risk was correlated with higher MFG-E8 levels. The HR was 0.81 (95% CI, 0.74–0.98, p < 0.003). PCT levels and APACHE II scores were not statistically associated with mortality (p = 0.709 and p = 0.078). The following is the Harrell C-index for mortality: MFG-E8, 0.79 (95% CI 0.72, 0.93); PCT, 0.68 (0.46, 0.89); APACHE II score, 0.59 (0.37, 0.75); and SOFA score, 0.845 (0.74, 0.91).

**Table 2:** Associations between each variable and mortality.

Variables	Univariate analysis		
	HR (95% CI)	p	Harrell C-index
MFGE8	0.81 (0.74, 0.98)	**0.003**	0.79
PCT	0.75 (0.16, 3.49)	0.709	0.68
APACHE II score	1.03 (0.73, 1.15)	0.078	0.59
SOFA score	1.12 (0.94, 1.21)	**0.002**	0.845

We used Cox’s proportional hazard model to evaluate associations between each variable and mortality.

*MFGE8* milk fat globule epidermal growth factor 8, *PCT* procalcitonin, *APACHE* Acute Physiology and Chronic Evaluation, *SOFA* Sequential Organ Failure Assessment, *HR* hazard ratio, *CI* confidence interval.

Significant values are in bold.

A 5-day survival study was conducted to further elucidate the connection between rmMFG-E8 and the severity of the disease. All septic mice died within 3 days after CLP surgery, while 40% of mice who received rmMFG-E8 treatment survived over 5 days (Fig. 1D). The results indicated that MFG-E8 could effectively improve the survival rate of septic mice.

### Treatment with rmMFG-E8 attenuated organ injury

**Figure 3:**
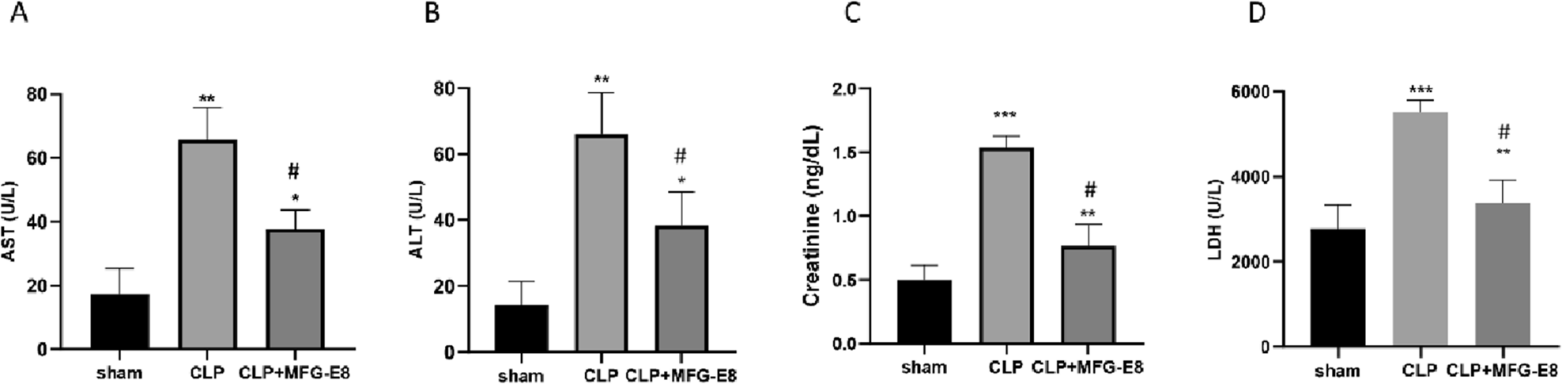
Treatment with recombinant MFG-E8 attenuates organ injury. Levels of AST (**A**), ALT (**B**), Creatinine (**C**), and LDH (**D**) were measured 24 h after CLP and found to be reduced in the rmMFG-E8 treatment group. Data are expressed as means ± SEM and compared by one-way ANOVA by Dunn’s and SNK method, and Mann Whitney Rank Sum test (*p < 0.05 versus sham group; ^#,^*p < 0.05 versus CLP group; *p < 0.05, **p < 0.01, ***p < 0.001).

Sepsis is thought to cause injury in multiple distant organs. Compared with sham mice, CLP-induced septic mice exhibited liver and kidney damage, including increases in AST (Fig. 3A), ALT (Fig. 3B) and Cr (Fig. 3C) by more than threefold and a 1.9-fold increase in LDH (Fig. 3D). In contrast, rmMFG-E8 treatment produced significant reductions of 25.8% in ALT (Fig. 3A), 30.3% in AST (Fig. 3B), 36.4% in Cr (Fig. 3C) and 29.5% in LDH (Fig. 3D) compared to the CLP group.

### Treatment with rmMFG-E8 reduces the levels of inflammatory factors in CLP-induced septic mice

**Figure 4:**
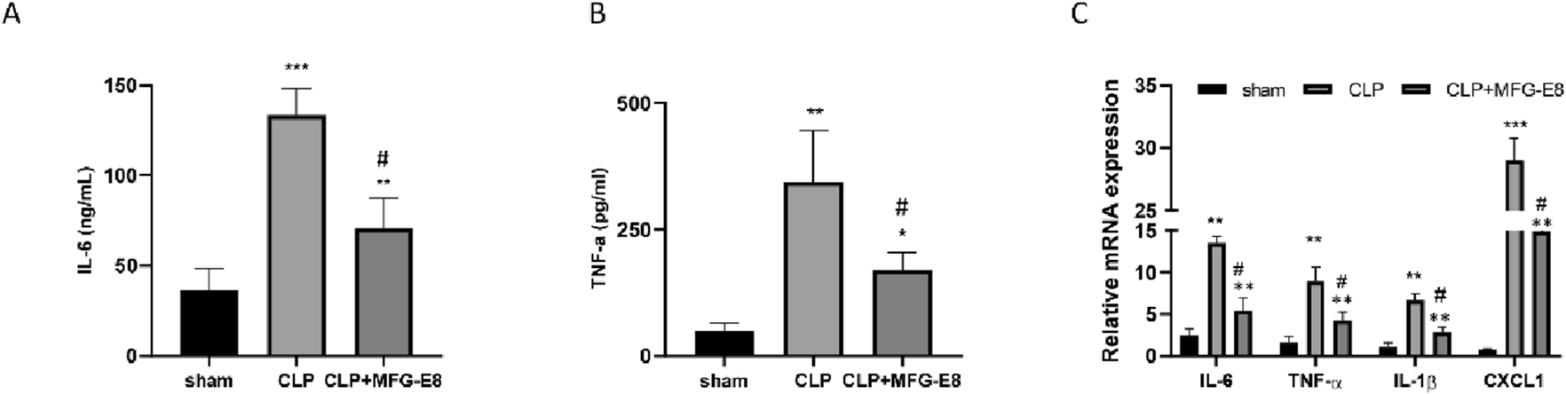
MFG-E8 administration reduces levels of inflammatory factors in the serum and liver tissues of CLP-induced septic mice. (**A,B**) Serum levels of inflammatory factors (IL-6, TNF-α) in septic mice at 24 h after CLP. (**C**) Relative mRNA expression levels of inflammatory factors (IL-6, TNF-α, IL-1β, and CXCL1) in the liver of septic mice at 24 h after CLP after normalization with GAPDH mRNA levels as determined by qRT-PCR. n = 6, mean ± SEM. (*p < 0.05 versus sham group; ^#,^*p < 0.05 versus CLP group; *p < 0.05, **p < 0.01, ***p < 0.001).

The effect of MFG-E8 in reducing the inflammatory response in patients with sepsis was evaluated by detecting the levels of inflammatory factors in serum and vital organs. The results implied that in septic mice, administration of MFG-E8 reduced serum IL-6 and TNF-α levels (Fig. 4A,B). After MFG-E8 treatment, the correlative mRNA expression levels of IL-6, TNF-α, IL-1β, and CXCL1 in the livers of septic mice were obviously reduced (Fig. 4C). Taken together, these results show that MFG-E8 inhibits systemic inflammation in septic mice.

### MFG-E8 suppressed ferroptosis in CLP-induced septic mice

Ferroptosis is related to cell redox balance, iron ions and oxidative stress, and the main driving factor is the peroxidation of cell membrane phospholipids^22^.

**Figure 5:**
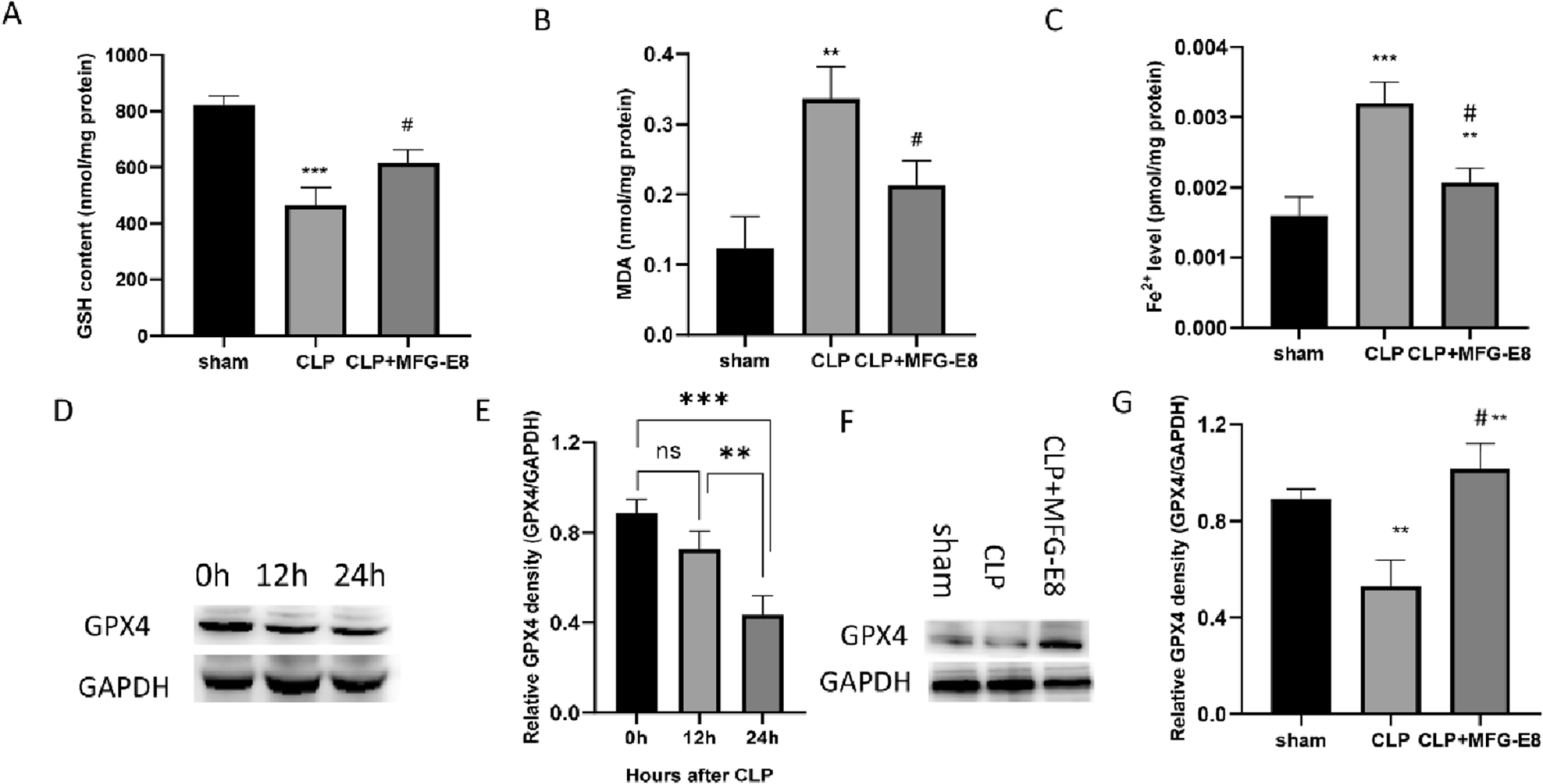
MFG-E8 suppressed ferroptosis in CLP-induced septic mice. Liver glutathione (GSH) contents (**A**), liver malondialdehyde (MDA) contents (**B**) and liver ferrous (Fe^2+^) concentrations (**C**) were measured 24 h after CLP. (**D,E**) Western blot analysis of GPX4 in the liver of CLP-induced septic mice at 0, 12 and 24 h after CLP. n = 3, mean ± SEM. (**F,G**) Western blot analysis of GPX4 in the liver of CLP-induced septic mice 24 h after CLP with MFG-E8 treatment. n = 3, mean ± SEM. (*p < 0.05 versus sham group; ^#,^*p < 0.05 versus CLP group; *p < 0.05, **p < 0.01, ***p < 0.001).

We assessed oxidative stress in CLP-induced septic mice. Sepsis decreased the production of GSH (Fig. 5A), an antioxidative marker. In addition, sepsis induced significant increases in MDA (Fig. 5B) and liver ferrous (Fe^2+^) concentrations (Fig. 5C), indicating higher oxidative stress. Treatment with MFG-E8 reversed the above changes caused by sepsis and appeared to diminish oxidative stress in the liver.

Glutathione peroxidase 4 (GPX4) is the key for maintaining cell lipid homeostasis and is negatively correlated with the levels of ferroptosis^23^. To assess ferroptosis in mice with sepsis, GPX4 was used as a measure of ferroptosis. The density of GPX4 in the liver decreased in a time-dependent manner after CLP was introduced (Fig. 5D,E). MFG-E8 treatment after CLP surgery increased GPX4 expression to a high level (Fig. 5F,G).

The results above demonstrated that MFG-E8 may have protective effects by inhibiting ferroptosis.

## Discussion

MFG-E8 has been considered a new treatment for improving the outcome of inflammatory disease, but the mechanism has not been clarified^24^. Prior studies showed that MFG-E8 suppressed oxidative stress in renal fibrosis^25^ and subarachnoid haemorrhage^26^. In this study, our purpose was to study the expression of MFG-E8 in patients with sepsis and its clinical significance.

We first observed that serum MFG-E8 levels were decreased. The serum level of MFG-E8 negatively correlated with the APACHE II score and PCT, which are markers of hospital mortality and infection^22^, indicating that MFG-E8 revealed the severity of sepsis. Furthermore, MFG-E8 levels were lower in nonsurvivors than in survivors among septic patients. MFG-E8 levels were also shown to be significantly associated with mortality, and MFG-E8 might serve as a useful prognostic biomarker for sepsis. Moreover, we also demonstrated that MFG-E8 exerted an anti-inflammatory effect by reducing ferroptosis. MFG-E8 reduced ferroptosis by activating GPX4, which might be an alternative treatment target for sepsis.

In our research, we found that serum MFG-E8 levels are decreased in septic patients. In-depth exploration of the reasons needs to be conducted. First, activated macrophages and immature dendritic cells, which are antigen-presenting cells, secrete MFG-E8^5^. Sepsis is marked by a systemic inflammatory response, which promotes the activation and maturation of antigen-presenting cells; during this maturation process, the production of MFG-E8 is reduced^27^. Furthermore, MFG-E8 has anti-inflammatory effects^8^, and whether MFG-E8 is consumed during the anti-inflammatory process needs further research.

Our study demonstrated that MFG-E8 levels are related to mortality. The index of predictive power, that is, the Harrell C-index, was 0.79 for MFG-E8. This C-index was better than the C-indices for the APACHE II and PCT scores (0.59 and 0.68) and was inferior to the C-index of the SOFA score (0.845). The SOFA score is equivalent to the APACHE II score in accuracy for prognosis among severely ill sepsis patients in the ICU^28,29^. Conversely, evidence-based recommendations have demonstrated that PCT is linked to mortality in septic patients and can moderately predict sepsis mortality^30^. In the current study, according to the C-index, we propose that MFG-E8 may be a better biomarker for prognosis in sepsis patients than the APACHE II score and PCT. The sample size is quite limited, and this is a retrospective study, so more research is required to verify the results.

Some research shows that MFG-E8 has anti-inflammatory effects. The direct anti-inflammatory properties of MFG-E8 are complex. First, excessively activated neutrophils are recruited into tissues, and the secondary necrosis of apoptotic cells may contribute to inflammation in sepsis. MFG-E8 promotes apoptotic cell engulfment by binding to phosphatidylserine (PS) on the cell membrane via its discoidin domains while also binding to αvβ3 integrin on macrophages and other phagocytic cells via its RGD motif (arginine-glycine-aspartic acid)^31–33^. Therefore, MFG-E8 reduces inflammation by promoting apoptotic cell binding to macrophages. In addition, NADPH oxidase-induced oxidative stress induces PS externalization in neutrophilic cells, facilitating their recognition by macrophages through MFG-E8 and accelerating the clearance of apoptotic cells^34^. Apart from its role in the engulfment of apoptotic cells, MFG-E8 also exerts a protective effect through other signalling pathways. MFG-E8 reduced lipopolysaccharide (LPS)-induced oxidative stress in the mouse brain through the Keap-1/Nrf-2/HO-1 pathways^12^. The protective effect of MFG-E8 was blocked by knockdown of integrin β3, indicating that integrin β3 is involved in Keap-1/Nrf-2/HO-1 pathways^26^. The suppression of antioxidant factors, such as NQO1, GSH, SOD1 and SOD2, by LPS was reversed by MFG-E8. However, MFG-E8 did not change the production of antioxidant molecules under normal conditions, indicating that MFG-E8 function was activated under certain circumstances^12^.

Ferroptosis is involved in multiple organ injuries and the inflammatory response of sepsis. Inhibition of ferroptosis may relieve the inflammatory response and improve organ functions^22,35^. MFG-E8 can regulate inflammation, and we infer that it may be involved in regulating ferroptosis. In our research, we observed a ferroptosis phenomenon in which MFG-E8 restored the density of GSH and GPX4 in the liver and attenuated the increase in the ferrous concentration in the liver. GPX4 is an essential regulator of ferroptosis that converts GSH into oxidized glutathione and turns cytotoxic lipid peroxides into the corresponding alcohols^36^. Cells with knockdown of GPX4 underwent cell death accompanied by lipid reactive oxygen species (ROS) generation, while overexpression of GPX4 inhibited ferroptosis. GSH is a vital cofactor of GPX4 in the process of hydrogen peroxide reduction. Several studies have reported reduced GSH and GPX4 as well as increased lipid peroxidation in bacterial infections^37^. Consistent with their results, we observed GSH depletion and a decreased density of GPX4 in our septic mice. Treatment with MFG-E8 reversed the reductions in GSH and GPX4. However, the mechanism of this reduction deserves further research.

Sepsis is a disease characterized by multiple organ injury. Our study mainly focused on the protective role of MFG-E8 in liver damage. Other research has shown that MFG-E8 attenuates organ injury of the kidney^38^, lung^19^ and spleen^39^ by anti-inflammatory effects or mediating the clearance of apoptotic cells. It is unclear on which organ MFG-E8 has the strongest protective effect. Our research is the first to suggest that MFG-E8 alleviates liver damage by regulating ferroptosis, and whether MFG-E8 alleviates other organ damage by regulating ferroptosis needs to be researched.

In conclusion, we confirmed that MFG-E8 might be a good biomarker for diagnosing sepsis and predicting patient outcomes. However, larger cohort studies are required to verify our findings. In addition, our results implied that MFG-E8 suppressed inflammation and ferroptosis in sepsis.

## Supplementary Information

Supplementary Figure 1.

Supplementary Tables.

## Data Availability

The datasets during and/or analysed during the current study available from the corresponding author on reasonable request.
