# Supplementary material for: MFG-E8 has guiding significance for the prognosis and treatment of sepsis
Source: Sci Rep. 2022 Dec 3;12:20916. doi: 10.1038/s41598-022-25601-8 (PMC9719492; doi:10.1038/s41598-022-25601-8)
Supplement: Supplementary file 1 — Supplementary Figure 1. [file 41598_2022_25601_MOESM1_ESM.doc]

**D**


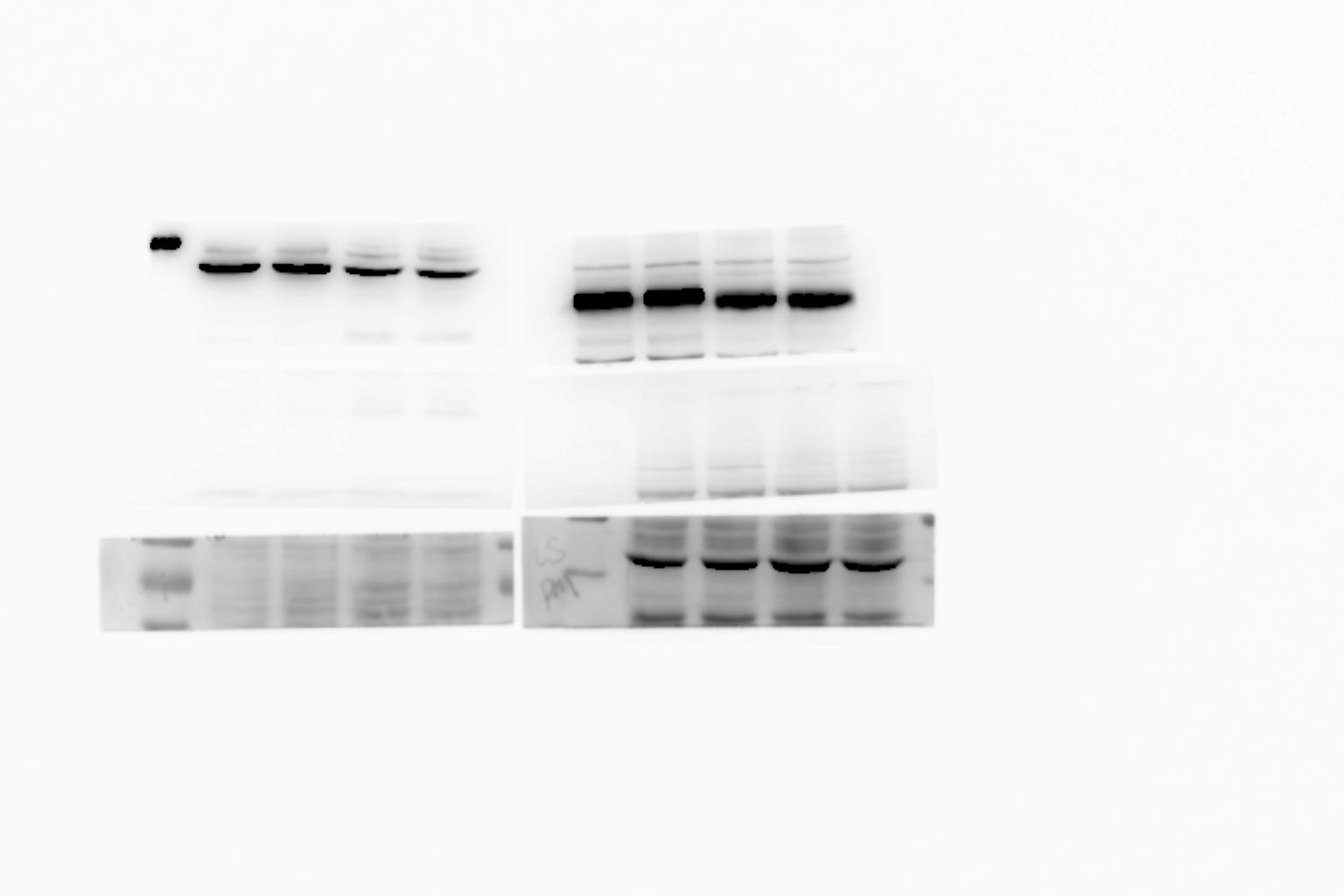


Con 0h 12h 24h

GPX4 17kDa

Con 0h 12h 24h

36kDa GAPDH


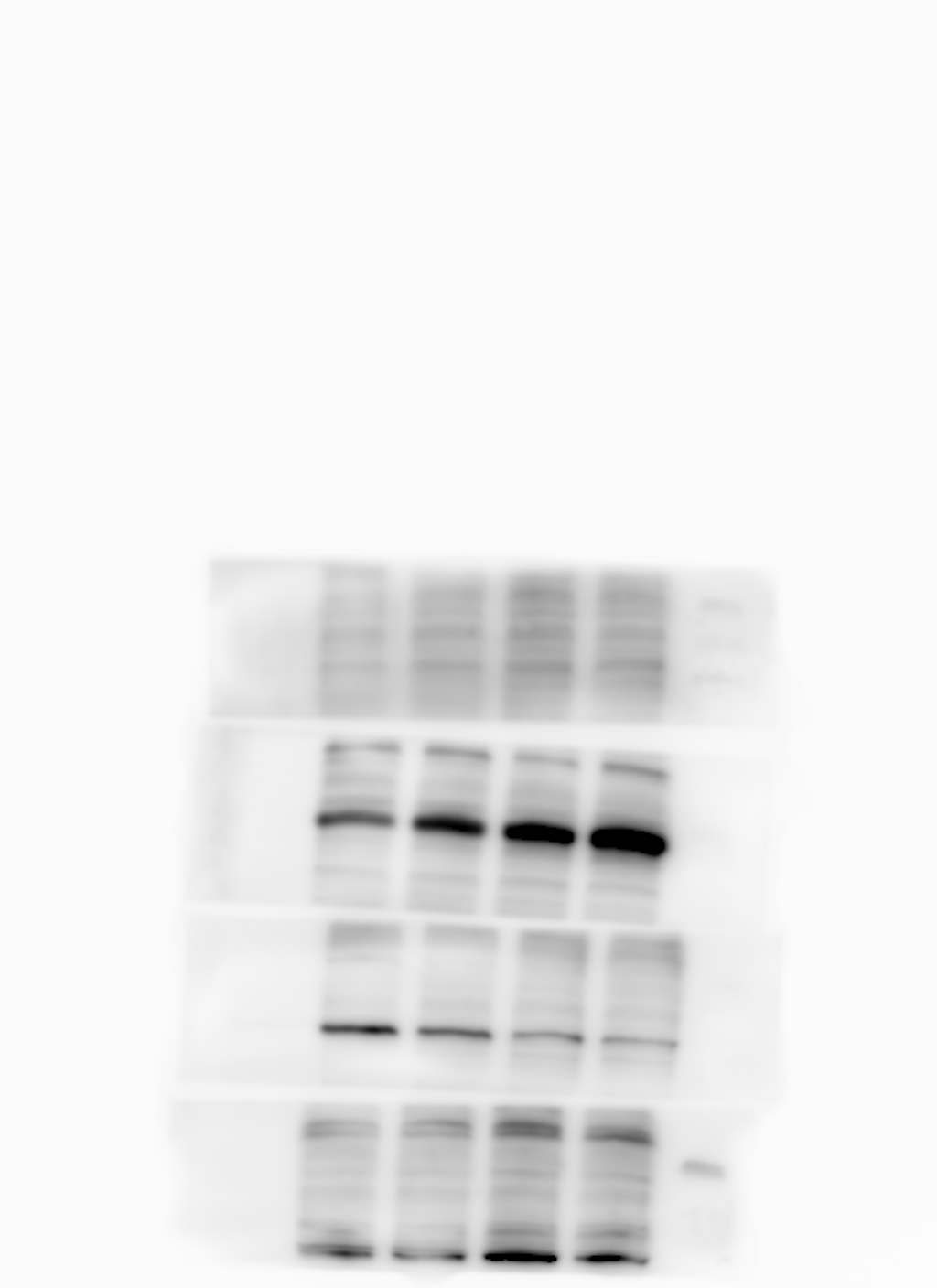
**F**

**
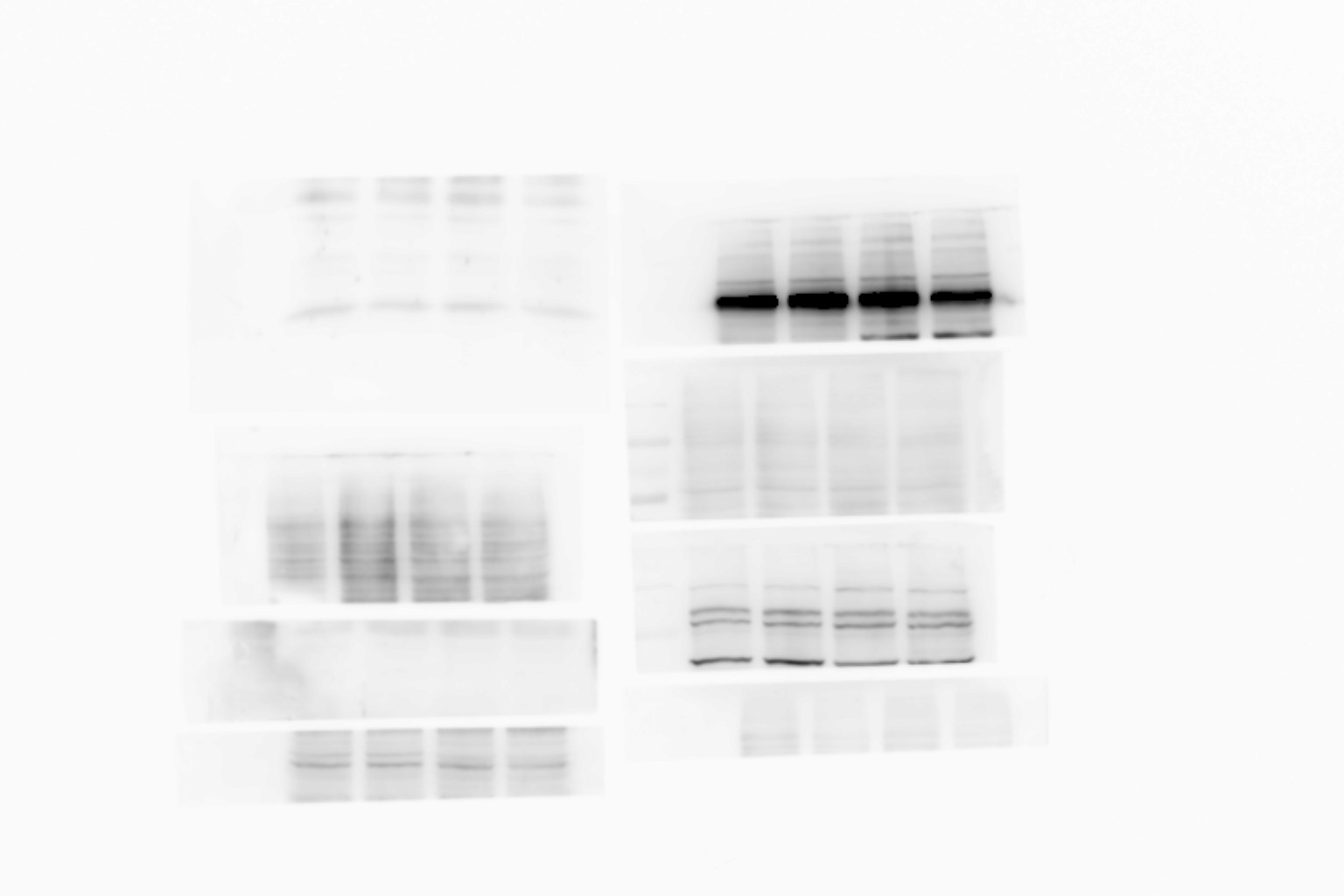
**

Sham CLP CLP+M Sham

GAPDH 36kDa

Sham CLP CLP+M Sham

GPX4 17kDa

**Supplementary figure 1.** The D and F are the original full-length blots for figure 5D and figure 5F.
