# Supplementary material for: MFG-E8 has guiding significance for the prognosis and treatment of sepsis
Source: Sci Rep. 2022 Dec 3;12:20916. doi: 10.1038/s41598-022-25601-8 (PMC9719492; doi:10.1038/s41598-022-25601-8)
Supplement: Supplementary file 2 — Supplementary Tables. [file 41598_2022_25601_MOESM2_ESM.docx]

Supplementary Table 1. Primers for qPCR analyses of the relevant sequences

| **Mice** | *CXCL1* | F: TGCACCCAA ACCGAAGTC  R: GTCAGA AGCCAGCGT TCACC |
| --- | --- | --- |
|  | *IL-1β* | F: GGGCCTCAAAGGAAAGAATCT  R: GAGGTGCTGATGTACCAGTTGG |
|  | *IL-6* | F: CTGGGAAATCGTGGAAATGAG  R: AAGGACTCTGGCTTTGTCTTTCT |
|  | *TNF-α* | F: GCC AGA GCCACATGCTCCTA  R: GATAAGGCTTGGCAACCCAAGTAA |
|  | *GAPDH* | F: AGTCGGTGTGAACGGATTTG  R: GGGGTCGTTGATGGCAACA |

Supplementary Table 2. Demographics of healthy volunteers and septic patients

| Variables | Sepsis (N=100) | Control (N =30) | | *p* value | |
| --- | --- | --- | --- | --- | --- |
| Demographics |  | |  | |  |
| Age (y, Mean ± SD) | 66.2±21.4 | | 58.1±17.0 | | 0.71 |
| Gender(male/female) | 47/53 | | 13/17 | | 0.63 |
| Laboratory results (mean±SD or median, interquartile range)  WBC (×10^9^/L)  Neutrophils (%)  Platelet count (×10^9^/L)  ALT (U/L)  TBIL (μmol/L)  Cr (μmol/L)  ALB(g/L) | 13.2±5.1  86.2±6.3  180.2±56  95 (2-6001)  35.2 (3.99-290)  109 (28-827)  32.5±3.9 | | 9.5±3.2  82±5.7  167.3±45  34 (7-530)  22 (3.5-258)  60.5 (17-680)  38.7±6.4 | | 0.44  0.38  0.064  0.062  0.48  0.064  0.76 |
| cTnT(pg/mL)  MFG-E8(ng/mL) | 478 (7-5139000)  5.60±3.80 | | 255(7-7240000)  11.74±5.44 | | 0.057  **＜0.001** |

Abbreviations: WBC, Leucocytes; cTnT, cardiac troponin T; NT-proBNP, NT-proB-type Natriuretic Peptide; ALT, alanine transaminase; TBIL, total bilirubin; Cr, creatinine；ALB, albumin; PCT, procalcitonin; MFG-E8, milk fat globule epidermal growth factor 8；

APACHE II score, Acute Physiology and Chronic Health Evaluation II score; SOFA score, Sequential Organ Failure Assessment Score

Supplementary Table 3. Significance of MFG-E8 for predicting mortality

| Cut off values  (ng/mL) | Sensitivity(%) | Specificity(%) | | Youden index | |
| --- | --- | --- | --- | --- | --- |
| 2.0 | 0.42 | | 0.94 | | 0.36 |
| 3.0 | 0.42 | | 0.81 | | 0.22 |
| 3.86  4.51 | 0.83  0.83 | | 0.71  0.61 | | 0.54  0.45 |
| 5.09 | 0.83 | | 0.55 | | 0.38 |
| 6.00  6.95 | 0.83  0.92 | | 0.43  0.31 | | 0.27  0.22 |
